# Patient and Public Acceptance of Digital Technologies in Health Care: Protocol for a Discrete Choice Experiment

**DOI:** 10.2196/46056

**Published:** 2023-08-10

**Authors:** Ann-Kathrin Fischer, Axel C Mühlbacher

**Affiliations:** 1 Department of Health, Care, Management University of Applied Sciences Neubrandenburg Neubrandenburg Germany

**Keywords:** health preference research, stated preference survey, discrete choice experiment, study protocol, digital transformation, digital technologies, digital interventions, health care, rehabilitation, stroke, mobile phone

## Abstract

**Background:**

Strokes pose a particular challenge to the health care system. Although stroke-related mortality has declined in recent decades, the absolute number of new strokes (incidence), stroke deaths, and survivors of stroke has increased. With the increasing need of neurorehabilitation and the decreasing number of professionals, innovations are needed to ensure adequate care. Digital technologies are increasingly used to meet patients’ unfilled needs during their patient journey. Patients must adhere to unfamiliar digital technologies to engage in health interventions. Therefore, the acceptance of the benefits and burdens of digital technologies in health interventions is a key factor in implementing these innovations.

**Objective:**

This study aims to describe the development of a discrete choice experiment (DCE) to weigh criteria that impact patient and public acceptance. Secondary study objectives are a benefit-burden assessment (estimation of the maximum acceptable burden of technical features and therapy-related characteristics for the patient or individual, eg, no human contact), overall comparison (assessment of the relative importance of attributes for comparing digital technologies), and adherence (identification of key attributes that influence patient adherence). The exploratory objectives include heterogeneity assessment and subgroup analysis. The methodological aims are to investigate the use of DCE.

**Methods:**

To obtain information on the criteria impacting acceptance, a DCE will be conducted including 7 attributes based on formative qualitative research. Patients with stroke (experimental group) and the general population (control group) are surveyed. The final instrument includes 6 best-best choice tasks in partial design. The experimental design is a fractional-factorial efficient Bayesian design (D-error). A conditional logit regression model and mixed logistic regression models will be used for analysis. To consider the heterogeneity of subgroups, a latent class analysis and an analysis of heteroscedasticity will be performed.

**Results:**

The literature review, qualitative preliminary study, survey development, and pretesting were completed. Data collection and analysis will be completed in the last quarter of 2023.

**Conclusions:**

Our results will inform decision makers about patients’ and publics’ acceptance of digital technologies used in innovative interventions. The patient preference information will improve decisions regarding the development, adoption, and pricing of innovative interventions. The behavioral changes in the choice of digital intervention alternatives are observable and can therefore be statistically analyzed. They can be translated into preferences, which define the value. This study will investigate the influences on the acceptance of digital interventions and thus support decisions and future research.

**International Registered Report Identifier (IRRID):**

DERR1-10.2196/46056

## Introduction

### Background

Strokes pose a particular challenge to the health care system. Although stroke-related mortality has declined in recent decades, the absolute number of new strokes (incidence), stroke deaths, and survivors of stroke has increased [1]. Approximately 40% of patients with stroke present with persistent cognitive, perceptual, and participative disabilities [2,3]. Stroke is the second leading cause of acquired disability [4]. Neurorehabilitation reduces disability and improves functional outcomes [5]. Stroke-related limitations are treated in neurorehabilitation. Most recovery of functions is achieved in the subacute phase (3-6 months after the stroke event) [6]. Neurorehabilitation is a combination of various disciplines [7]. Because of the need for a comprehensive therapy approach, strokes are one of the most cost-intensive diseases in the German health care system, with current treatment costs of approximately €43,000 (US $48,040.40) per patient [8].

### Objectives

With the increasing need of neurorehabilitation and the decreasing number of professionals, innovations are needed to ensure adequate care [9]. Digital transformation has opened avenues for improving services, products, and interventions through the increased use of digital technologies [10,11]. In the project *evidence-based robotic assistance in neurorehabilitation* (E-BRAiN), a robot was developed to support daily therapy practice [12]. Researchers investigated the use of the humanoid robot “Pepper” by SoftBank Robotics owing to interaction [13], motivation, software and hardware capabilities, and acceptance. To decide on implementing innovative therapies, decision makers should consider the trade-offs that patients are willing to accept [14]. Discrete choice experiments (DCEs) are a way to identify trade-offs by analyzing preferences [15]. The acceptance of new therapies is a key success factor and is determined by the fulfillment of needs [16-18]. When acceptance information is considered in decision-making, the utility of care decisions can be maximized in the short term [19,20], can strengthen patient orientation and adherence in the intermediate term, and can improve clinical effects in the long term. Financially, the health care system can benefit from faster and more efficient therapeutic outcomes [21,22].

## Methods

### Ethics Approval

The preference survey instruments, informed consent form, and study design were reviewed and approved by the ethics committee of Hochschule Neubrandenburg (HSNB/177/21). The study considers the Declaration of Helsinki, the Deutsches Bundesdatenschutzgesetz (the German Data Protection Act), the European privacy policy, and Guideline on Good Clinical Practice (CPM/ICH/135/95) regarding the obligation to report serious adverse events.

### Informed Consent, Participation, and Dissemination

Participant confidentiality was ensured by pseudonymization of the qualitative interview recordings and will be ensured by anonymizing the data from the quantitative survey (DCE). All participants have provided informed consent in the completed qualitative interviews. Informed consent is also obtained in the quantitative survey. Participants have the right to withdraw from the study at any time and without giving reasons and to request the deletion of the collected data at any time during the survey. All participants will be informed on the opening page of the web-based questionnaire that participation is voluntary. All identifiable details will be removed during future dissemination of findings and in presentations and publications. Researchers’ contact information is provided on the last page in case the participants have questions. The participants can contact the researchers directly by phone or via a contact email address. The results of this study will be presented at national and international conferences and published in peer-reviewed journals.

### Research Questions and Study Aim

This ongoing study will address the following question: Which criteria of digital technologies impact patient and public acceptance? The aim is to investigate patient and public preferences to generate information on acceptance and analyze the value of digital interventions. Preference evidence is a series of observable human behaviors that can be analyzed by the choices individuals make between alternative health states, health services, or health interventions. Choices define the value of attributes and alternatives in health care.

Acceptance is a requirement for active patient engagement and thus for efficiency (the greatest possible result with the least possible effort) and effectiveness (ability of the intervention to positively improve patients’ state of health in a targeted manner). To obtain information on acceptance, it is necessary to understand the trade-offs that patients make by weighing the advantages and disadvantages of therapy options using digital technologies. We also ask whether patients would accept a humanoid robot in neurorehabilitation. Our primary objective is to weight the criteria that impact patient and public acceptance. Secondary objectives include (1) a benefit-burden assessment (estimation of the maximum acceptable burden of technical features and therapy-related characteristics for the patient or individual, eg, no human contact), (2) overall comparison (assessment of the relative importance of attributes for comparing digital technologies), and (3) adherence (identification of key attributes that influence patient adherence). Our exploratory objectives include (1) heterogeneity assessment (how preferences differed based on participant characteristics) and (2) subgroup analysis (to explore and explain heterogeneity based on the correlation between therapy preferences and participants’ sociodemographic information, experience-based treatment history, and attitudes). Our methodological aims are to investigate the use of DCE and the impact on data quality, preferences, and choice consistency.

The following assumptions and hypotheses will be examined:

1. Patients engage in a health care process to meet their needs. Health-related needs include therapy outcomes. Therapy outcomes can be measured by goal achievement. Therapy outcomes influence the choice of intervention.
  - H1: The higher the expected success rate, the higher the probability of choosing an alternative.
  - H0: The success rate of an intervention has no influence on the probability of choosing an alternative.
2. Needs involve technical aspects of digital technologies. Undesirable features will have a negative impact on the decisions.
  - H1: The more burdensome or worse a technical aspect is rated, the lower the probability of choosing an alternative.
  - H0: The rating of a technical aspect has no influence on the probability of choosing an alternative.
3. The complete absence of human contact has a negative effect on the decision.
  - H1: The fewer the opportunities for human contact, the lower the probability of choosing an alternative.
  - H0: The opportunity for human contact has no influence on the probability of choosing an alternative.
4. Digital technologies should have certain characteristics according to the goals of a digital transformation (eg, flexibility, adaptability, participation, and empowerment). Characteristics that do not condition flexibility, adaptability, participation, or empowerment negatively impact patients’ therapy decisions.
  - H1: The fewer the goals of the digital transformation that can be achieved by the digital technology, the lower the probability of choosing an alternative.
  - H0: Flexibility, adaptability, participation, and empowerment have no influence on the probability of choosing an alternative.
5. Interventions should be individualized for patients in a patient-centered health care system. Patients’ preferences differ with respect to their characteristics (sociodemographic characteristics, clinical and digital experiences, attitudes, expectations, norms, behaviors, and abilities).
  - H1: The less patient centered an intervention is, the lower the probability of choosing an alternative.
  - H0: The characteristics of different patients have no influence on the probability of choosing an alternative.

### Target Population

We include two study populations: (1) an experimental group (patients with stroke) and (2) a control group (the general population). Only individuals diagnosed with a stroke will be included in the experimental group. For both groups, only individuals aged ≥18 years, who reside in Germany, and who can read and understand German will be included. Limitations in recruitment were expected because of the COVID-19 pandemic at the beginning of the study and during the development of the experiment. Therefore, we decided to include a control group.

### Overall Project Design

Both qualitative and quantitative methods are used in this health preference research (HPR) study. A completed qualitative prestudy was used to identify which decision criteria patients consider when choosing innovative therapy options using digital technologies. Furthermore, the impact of these attributes will be determined in a quantitative survey using a DCE. DCE is a stated preference method for economic evaluation [23,24]. This DCE will be used to analyze how patients define the “value” of a digital technology. The analysis is performed using repeated choices between hypothetical therapy alternatives with different attribute levels [25]. The choices can identify which attributes and levels are preferred. Patient benefit derives from preferences, and thus, the benefit can also be defined as value [26]. DCE is a preference elicitation method [27-29]. The use of such choice experiments is also presentive in medicine [30,31]. There are many guidelines and recommendations for the DCE [32,33]. The increasing popularity of DCE reinforces the intention in this study. This paper describes the protocol of the DCE to investigate patient and public acceptance of digital technologies in health care. This paper also describes the methodology used in our ongoing work.

### Relevance of the DCE

A targeted literature research on acceptance models was conducted to analyze acceptance dimensions and criteria that impact individual’s acceptance. Criteria from different perspectives, such as those of the patients, system, and provider, were analyzed to investigate the concept of acceptance with respect to the value of digital interventions. Acceptance refers to the willingness of individuals to engage in a health intervention or health service [34]. Technology acceptance models provide information on variables that influence acceptance, such as perceived usefulness, perceived ease of use, subjective norms, or user experience [35-37]. Extensions of the models explain the interrelationships between the beneficial influencing factors and the burdensome influencing factors as a balancing process [38]. However, the models are limited in evidence regarding specific attributes that represent a benefit or a cost or burden to individuals or, specifically, patients. No information is provided on how impactful individual aspects of digital information are. HPR studies were conducted to generate evidence regarding the weighting of attributes. Thus, the presented DCE was developed.

The aspects of digital technologies (eg, data processing) have already been examined in various studies. There are studies that focus on digital apps on mobile devices [39,40]. Among these studies, attention is partly focused on data processing only [41,42]. Other studies examine factors of communication (eg, information giving, information transfer, and contact) [41-43]. Most of the studies do not include health-related goal variables [39,44]. Thus, there is a gap in holistic evidence on preference research on digital interventions. This study aims to generate evidence for all possible digital technologies (not only digital apps).

### Development of Attributes and Levels

Different digital technologies have been investigated in different studies in the context of therapies and were therefore considered in the prestudy (robots, voice assistants, digital health apps, digital health devices, etc). As digital technologies have an unlimited set of attributes, limitations arise from the fact that decision makers cannot consider all possible attributes in their evaluation [18]. In a DCE, alternatives are described by decision-relevant criteria that are identified from the set of possible attributes [18].

A targeted literature search was conducted to identify the characteristics of health-related interventions in the context of the digital transformation associated with the use of digital technologies. First, digital transformation in health care was considered. Digital transformation became essential due to the rapidly changing and increasing needs and expectations in health care [45-47]. Digital technologies are innovations to achieve certain goals of digital transformation: flexibility of place and time, flexibility of the range of care, expansion of access for patients considering gains and pains, improvement of health literacy, participation and personal responsibility of patients, and improvement of information structures [11,48]. Certain characteristics (attributes) of digital technologies are necessary to achieve these goals. In total, 17 attributes were identified in the literature: communication, emergency communication, contact with professionals, distance to technology, emotions, speech, participation, recognition, feedback, data security, pick-up and drop-off function, entertainment, usefulness, place, location dependency, duration, and frequency.

Formative qualitative research is foundational to the development process of HPR studies [49]. Therefore, we conducted semistructured pilot interviews with patients with stroke (n=14) and experts in neurorehabilitation (n=5) to identify and cluster the decision-relevant criteria from the subset of 17 attributes. Patients were asked to describe their physical and daily living limitations owing to the diagnosis and their experiences with neurorehabilitation. The patients were also asked about their attitude, opinions, and expectations regarding digital technologies. After the humanoid assistance robot “Pepper” was presented, the participants were asked about their attitude toward the robot. Attributes defined from the literature were presented with descriptions on cards. The patients were asked to rank the attributes according to their relevance. Furthermore, the patients described what they thought a future therapy might look like by using a different card of attributes. An interview guide was used for this process. We used semistructured interviews, which allow an open discussion style and an interview to be individualized, allow spontaneous in-depth discussion of interesting statements, and allow a return to relevant key questions [50]. Experts estimated the patients’ point of view regarding the questions asked according to their experience. A list of seven decision-relevant criteria was developed: (1) explanation and presentation of exercises, (2) information from digital technologies, (3) contact with professionals, (4) patients’ choice in the therapy process, (5) data processing, (6) copayment per month, and (7) therapy success within 6 months. Thus, the list included 5 attributes that are technical aspects (attributes 1-5). In addition, copayment per month and therapy success within 6 months complete the decision context. Therapy success refers to an individual’s goal attainment. Goal attainment is determined by the improvement in body function, which in turn leads to a resumption of activities of daily living. The full list was concretized through discussion rounds, such as focus groups and workshops with experts in health care. The final list was transformed into a decision model.

### Descriptive Framework

Preference evidence can be identified by observing human behaviors using elicitation tasks. In a preference elicitation task, participants evaluate assigned alternatives or objects according to their preferences. In this study, we determined that the preference elicitation task would lead to the goal of determining a patient’s willingness to accept the trade-offs between the characteristics of therapy using digital technologies. The descriptive framework was created to contain all decision-relevant attributes, levels, and descriptions of the decision context that will be used to communicate the content of the elicitation tasks (Table 1).

**Table 1:** Descriptive framework.

Attributes	Description	Level 1	Level 2	Level 3	Level 4	Level 5
Explanation and presentation of therapy exercises	Patients receive information, instructions, and assistance on how to perform the exercises, with an explanation and presentation of therapy exercises.	Sounds and speech	Descriptive texts	Images	Videos	Spatial movement
Information in therapy	Patients receive information from the digital support system in digital therapies, but there are also digital therapies where patients do not get any information.	No information	Therapy and rehabilitation process	Diagnosed disease	Patient’s current health status	Change in health status owing to therapy
Contact with health care professionals	Patients have contact with health care professionals in a digital therapy, but there are also digital therapies in which patients have no contact with professionals.	No contact	Contact is indirect (messages)	Contact is direct (telephone or video)	—^a^	—
Patients’ choice in the therapy process	Patients have certain choices in a digital therapy. Therefore, patients have an influence on the design of therapy processes, but there are also therapies where patients have no influence.	No influence	Selection of therapy exercise with a certain degree of severity	Pace of therapy exercise	Time of therapy (frequency, duration, and start)	Place of therapy (eg, home, clinic, or practice)
Data processing	There is data processing in digital therapies. Data about patients are collected and forwarded, but there are also digital therapies where no data are processed.	No data processing	Processing of data about the person	Processing of data about the diagnosis	Processing of data about the progress of the therapy	—
Copayment per month	Patients in digital therapies contribute to the cost of the therapy with a copayment per month. There are also digital therapies where patients have no copayment.	€80 per month	€60 per month	€40 per month	€20 per month	No copayment
Therapy success within 6 months	Patients have success in a digital therapy by achieving their therapy goals. Therapy goals are set at the beginning of a therapy, but not all patients achieve their goals within 6 months.	60 out of 100 patients	70 out of 100 patients	80 out of 100 patients	90 out of 100 patients	100 out of 100 patients

^a^The number of levels of each attribute is not necessarily equal, so the number varies between 3 and 5 levels. Therefore, for the attributes contact with health care professionals, no data are available for levels 4 and 5, and for the attribute data processing, no data are available for level 5. Furthermore, the respondents in the study were recruited in Germany. Accordingly, the data on the copayment per month were given in euros (€) and not in US $ (€0.92=US $1). For visual representations of each level, see Multimedia Appendix 1.

### Choice Context

In choice experiments, participants are asked to assume that their therapy decision is made in the context of a specific or their current health condition. Our study sample included both therapy-experienced and therapy-inexperienced participants. Therefore, the participants are asked to assume a health condition that requires rehabilitative therapy.

### Elicitation Tasks

The challenge in a DCE is to dissolve the response uncertainty and to trace the preference information back to the individual attributes through repeated choice tasks, with participants “forced” to make a decision between digital therapies. For the professional group, there was no choice available within the choice task. The underlying choice task is a ranking task, which follows a fixed procedure. The choice starts with the selection of the best alternative among 3 choices. The choice is iterated with the remaining 2 so that a complete ranking is achieved. Thus, the DCE uses a best-best choice format [51].

Participants’ willingness to choose the digital therapy was determined by the following follow-up question: “Would you really choose the previously selected digital therapy in real life to improve your health?” Recently, the dual response “none” has been established as a variant in choice experiments [52,53]. A dual response of “none” asks for 2 responses for each task: one choice between the available product alternatives and one between the “none” concept and the previous alternatives. Essentially, it is a choice between the most preferred alternative and none. If participants would actually buy or take the alternative, they would not prefer none, but if they would not buy or take the alternative, they would prefer none to the given alternative [54]. The dual response “none” design takes more time to answer and is an increase in analysis effort owing to a complex design. However, more information is generated, which makes the design more efficient and powerful. Estimation errors are reduced, and the underlying model estimate is not significantly changed [52]. This design provides a higher resolution for individual-level preferences. As there is less “missing” information when participants select “none” as their preferred alternative, more information is available for individual-level modeling [54]. The final survey instrument comprised 6 choice tasks. The number of choice tasks was determined by the underlying experimental design, possible number of participants, and information received per task. No names, brands, or companies were named in the presentation of the choice tasks; therefore, the tasks were unlabeled. In addition, unlabeled alternatives were preferred because the study objectives were to estimate the marginal rates of substitution and value equivalents for therapy attributes [18].

### Elicitation Format

Participants will be familiarized with the attributes and expressions through descriptions and explanations before the first task. In addition, participants will see a tutorial and exercises to answer the choice tasks. Participants will answer a choice task with a clearly dominant alternative (copayment=none; treatment success=100%) so that an understanding of how to answer the DCE is tested.

### Experimental Design

Proper design and implementation of the DCE requires the consideration of the choice context, composition of choice sets, and framing of choice questions and instructions [55]. The results of the preliminary study indicated lower attention and concentration abilities in the experimental group. This finding, along with the evaluation of participants, led to the decision to use a partial design (choice tasks include 4 of the 7 attributes) [18,56]. Experimental design software was used in this study (Sawtooth Software Inc; ChoiceMetrics).

The underlying experimental design is a fractional-factorial efficient Bayesian design (D-error) to maximize the ability to identify participant preferences and variability in preferences across participants [56].

The experimental design was based on the following assumptions: minimal overlap, level balance and orthogonality. *Minimal overlap* means that each level is shown as few times as possible in a task. If the number of levels of an attribute is equal to the number of concepts in a task, each level is shown exactly once. Assumptions also were *level balance* which means that each level of an attribute is shown approximately the same number of times. *Orthogonality* means that levels are chosen independent of other levels so that the benefit of each level can be measured independent of all other effects).

Owing to the complexity of the experimental design in terms of the size of the decision model (7 attributes and 3-5 levels), the experiment was blocked. Combinations of alternatives are drawn from the total selection in 20 blocks in such a way that each level is combined as often and as evenly as possible. The study will analyze both the main and interaction effects. By combining all possible levels with each other (2-way frequency), sufficient information is generated to be able to measure the interaction effects. The allocation of the blockers is randomized. When a survey is stopped and choice tasks are not completed, they are made available to other participants so that all choice tasks are provided.

### Survey Instrument Design

Designing an effective survey instrument requires numerous connected decisions that are built on the decision context, attribute selection, and experimental design. The DCE requires a survey instrument that (1) explains to participants the various aspects of digital technologies that may differ across therapy options and (2) asks participants to indicate their preference for therapy options as aspects change. The final instrument is web based and self-completed by the participants.

The information presented to participants was developed by preparing descriptions for each attribute and level. Patients with stroke often experience cognitive impairment. Therefore, special attention must be paid to the layout of the survey. The design of the survey and the potential layout of the choice task should include the appropriate use of text and icons. An appropriate and well-designed layout can minimize the cognitive efforts required to compare alternatives and evaluate trade-offs. Because of the visual perceptual deficits in patients with stroke, icons should have good color contrast, clearly delineated lines, and a monochromatic design. We assessed the clarity and completeness of the DCE survey instrument and tested different choice formats (selection from 2 or 3 alternatives, best vs best-worst selection, and full vs partial design).

We limited the time required to complete the survey to a maximum of 30 minutes to minimize dropouts and to reduce data quality toward the end of the survey. First, we administered the quantitative survey with the final survey design in the control group. A pilot test was conducted, and the data collection was stopped after 150 completed questionnaires in the control group. Preference data were tested using a conditional logistic regression model to check for appearance validity, assumed signs and coefficients, and estimation problems.

We planned 3 sections for the web-based questionnaire: a study description and introduction to the attributes, the DCE, and the question section on sociodemographic and therapy and technology experience. The content of the survey will be clarified through an introductory description of the DCE survey technique at the beginning of the questionnaire. Here, participants will learn that they will be presented with hypothetical therapy alternatives in various combinations and that each alternative is described by 7 attributes. The questionnaire is expanded with questions on sociodemographic factors (including for inclusion and exclusion), sociocultural background, therapy-related and technology-related experiences, and health and quality-of-life assessment [57]. The questionnaire also includes a questionnaire assessment and questions on digital health literacy [58]. Different questions are designed to analyze the cognitive and perceptual limitations of the experimental group (Textbox 1). Metadata (or paradata) will provide information about the content of the health preference data. These data include the time taken to complete the survey and each question as well as information on dropout rates. The coding software used was Survey Engine GmbH. Textbox 1 presents the structure and content of the survey questionnaire.

**Structure and content of the survey questionnaire.**

**Introduction to the survey: data protection and technical instructions**

- Information on data protection regulations
- Information on responding via tablet and computer or laptop (exclusion: smartphone or iPhone)
- Information sheet for participants (PDF download)
- Declaration of consent for participants (as PDF download)
- Obtaining consent
- Instructions on how to respond to the survey, introduction to question formats, and options for saving an intermediate response status

**Testing of cognitive abilities and perception**

- A total of 5 picture and text questions (picture recognition, object naming or assignment, temporal or spatial orientation, and memory)
- Identification of cognitive and perceptual limitations with possible influence on the questionnaire response
- Basis: standardized assessments for identification of aphasia, apraxia, visual or perceptual deficits, and cognitive deficits

**Introduction to the survey: background of the digital stroke rehabilitation**

- Information about the diagnosis of stroke (incidence and shortage of specialists)
- Information about digital technologies (ie, digital support systems) in therapies
- Introduction of terms (digital support systems and digital therapies)
- Information about patient and public preferences, survey content (preference information, experiences, expectations, attitudes, and sociodemographic data), and survey process and structure

**Inclusion and representativeness of the study population**

- Inclusion: age <17 years, German language skills=intermediate to very good, and, only for experimental group, diagnosis of stroke=is present
- Representativeness in control group based on quota in age, sex, education, and state (for state, not present as quota in sampling)

**Diagnosis-related questions**

- Objective: to separate the study populations into patients with stroke and the general population
- Diagnosis of stroke; time of diagnosis; consequences of stroke related to physical functions, daily life, and support needs; and current treatment needs

**Introduction to the preference survey**

- Description of the scenario: rehabilitative therapy using digital technology
- Description and explanation of digital therapies (wording: digital support systems)
- General definition and delimitation of the terms attributes and attribute level
- Description and illustration of all 7 attributes and attribute expressions
- Example or tutorial for answering a choice set

**Preference elicitation or discrete choice experiment**

- Dominance test: selection from a choice set with an openly dominant alternative (no copayment and 100% treatment success) to test the understanding of choice tasks and quality (validity)
- 6 Choice tasks: choice best, choice second best, and dual response

**Additional questions**

- Evaluation of therapy goals as activities in daily life via a 5-point Likert scale
- Assessment of digital transformation goals in terms of flexibility of place, time, performance, and adaptability via ranking
- Sociodemographic questions (expanded) on marital status, household or residential situation, and occupational status
- Health status and quality of life
- Rehabilitative therapy experiences
- Experiences with and attitudes toward digital technologies for one’s own health
- Questionnaire assessment to evaluate the quality of the survey
- Questions on digital health literacy

**Completion of the survey**

- Acknowledgment
- Information on how to contact the study team and collaborators

### Statistical Analyses

Discrete choice models differ from regular logistic regression because the data are grouped. These models consider the structure of the DCE data, with the same participant providing multiple results for a sequence of different election scenarios [59].

We analyzed the results using conditional logistic and mixed logistic models and performed a latent class analysis to identify heterogeneous preferences [60]. We used the conditional logistic model to calculate the coefficients whose signs indicated a preference direction. Positive coefficients indicated a positive influence of a trait expression, and negative coefficients indicated a negative influence. The larger the coefficient, the greater the influence on the choice decision. The distances between 2 property expressions indicated the magnitude of the influence. The assumption of normal distribution in the conditional logistic model limited the informative value of the calculated coefficients. We used mixed logistic models to analyze preference differences as a function of each individual by calculating SDs and SEs. We tested assumptions to determine whether the preferences of specific groups differed from each other in terms of characteristics, experiences, and cultural background. Latent class analysis was used to generate information on the heterogeneous groups. We used StataCorp LLC to conduct our statistical analyses.

For our analysis, the results from both groups were compared. We did not directly compare the coefficients because of scale effects [61]. The comparison was made by considering the influence of the covariate general population.

### Study Sample

We include study participants depending on the target population, sample selection, and the qualitative or quantitative study part of the survey. The quantitative pilot interviews were conducted between March and September 2020. The qualitative pretest interviews were conducted between October and December 2021. The quantitative survey was started by pilot testing (n=150) in January 2022. After pilot testing, participants of the general population will be recruited via a panel provider and patients with stroke will be recruited via a panel provider, E-BRAiN clinical study and Stiftung Deutsche Schlaganfall-Hilfe, and from social networks. The study sample is based on a nonprobability ad hoc sample selection as a self-selection sample depending on availability and on a nonprobability ad hoc sample selection as a quota sample [62,63]. Regarding the full E-BRAiN project, recruitment and in-person participation of patients with stroke were planned within the clinical project. The DCE project was planned in relation to the clinical recruitment and was adjusted according to the pandemic-related constraints and financial resources. Therefore, panel recruitment was expanded to include patients with stroke in the DCE project.

### Data Collection

Calculating the sample size for the choice experiments is complex [64]. The question format, complexity of the choice task, desired precision of the results, and need for subgroup analysis are criteria that could modify the sample size [29,65].

For a robust quantitative evaluation of the main effects of a DCE in a simple logistic regression model (eg, mixed logit regression model or conditional logistic model), we calculated the sample size using the following formula: *(n* *× t × a) / c ≥1000; n = (1000 × c) / t × a*, where *n*=number of participants, *t*=number of choices (choice sets), *a*=number of choice alternatives per choice set, and *c*=number of levels (for main effects, *c*=number of levels of the attribute with the highest number of levels and for interaction effects, *c*=the largest product of the levels of the 2 attributes with the highest number of levels).

Given 6 choice tasks and 3 alternatives described by attributes with a maximum of 5 levels, a minimum number of 300 participants (*n = [1000 × 5] / [6 × 3]*) is needed to represent at least the main effects approximately 1000 times. This resulted in a target sample size of 300. When considering the interaction effects (*c*=15 in the formula), the resulting *n* is at least 1000 (*n = [1000 × 15] / [6 × 3]*). To adequately account for interaction effects, a sample size of at least 1000 is required to ensure sufficient representation. The final target sample size is approximately 1000 participants.

### Data Quality

Our data quality criteria are as follows. First, we require data security. The web-based survey is developed and scripted by the research team. Quality assurances are also conducted. The quality tests follow a predefined test grid. Second, we ensure data integrity by conducting several quality assurance steps, including extensive end-to-end testing, data export and tabulation of test data before the live sample will be launched, validation of the experimental design scripts, and validation of the data analysis scripts. Third, our data require validity and reliability. The following indicators of validity and reliability are collected: completion of the entire web survey in a short period, selection of the predominant alternative in the fixed-choice question, selection of a different alternative in the repeat question, selection of “best” and “second-best” levels always based on a single attribute, straightlining (ie, always the same answer choice in each choice set), and additional questions to verify DCE data and class determination.

## Results

The literature review, qualitative preliminary study, survey development, and pretesting were completed. Recruitment of the experimental group and the control group started in January 2022. Recruitment of the experimental group (patients with stroke) will be conducted in conjunction with the clinical trial of the collaborative project E-BRAiN. All patients in the clinical trial will be surveyed. Data collection and analysis will be completed in the last quarter of 2023.

## Discussion

### Principal Findings

Acceptance is a multidimensional construct. The requirements for digital technologies include various dimensions [66]. Digital technologies in postacute rehabilitative therapies are increasingly used for patients classified as cognitively, perceptually, and participatively impaired. [67]. These technologies are still largely unknown to users. Successful implementation depends on the acceptance of the patients [18]. In addition to expected clinical success, technical features also affect consumer acceptance.

This paper describes the development of a HPR study. Patient preference information will be provided by analyzing 7 attributes in a DCE. With respect to the mantra of HPR that “choice defines value,” the study objective is to weight criteria that impact patient acceptance. Furthermore, a benefit-burden assessment, an overall comparison of different alternative digital interventions, interpretation of adherence, analysis of heterogeneity, and subgroup analysis are aimed at improving decision-making for innovative interventions, such as adopting new digital technologies such as artificial intelligence (AI). This study includes several aspects that further test the hypotheses related to needs, outcomes, technical aspects, human contact, goals of digital transformation, and personal information about patients.

To achieve the objectives, formative preliminary studies were conducted. A list of 17 attributes was tested. The final list of attributes represents the decision context of digital transformation in health care: explanation and presentation of therapy exercises, information in therapy, contact with health professionals, and data processing. To complete the decision context, copayment per month and therapy success within 6 months were added.

To develop the final survey instruments, attributes were described and visualizations for each level were added in a descriptive framework. The developed fractional-factorial efficient Bayesian (D-error) experimental design includes a best-best ranking task in a partial profile. A web-based questionnaire was developed by integrating additional questions on patients’ characteristics, experiences, and cultural background. Pretest interviews and pilot testing have led to the holistic adaptation of the survey instrument in terms of descriptions, visualizations, and questions to enhance patient understanding as well as the testing of the experimental design. The analysis, with the help of a conditional logit model, proved the assignment of substantially results and unambiguous β coefficients to the attributes and levels, thereby demonstrating the effectiveness of the experimental design. The further study will use mixed logit models and heterogeneity analysis to evaluate the final study results.

This study demonstrates the importance of investigating patient acceptance and the technical aspects of digital technologies such as AI. Dai and Tayur [68] highlighted in their research that the uniqueness bias is a barrier to patient acceptance, as patients attribute more individuality to a human-guided therapy than to an AI-based solution. To solve this bias, a study of patient perceptions and preferences is recommended [68]. Furthermore, research on rhetoric to improve the adoption of digital technologies such as AI shows the importance of communicative strategies [69]. Another aspect that influences patient acceptance is data privacy and security and the associated risk [70].

To the best of our knowledge, this HPR study conducted a DCE. We attempt to develop a generic model that generates preference information to provide information on patient acceptance of digital technologies.

### Limitations

Further investigation must consider the following limitations and challenges:

- Digital transformation in health care is characterized by a large number of attributes to achieve goals (eg, flexibility). The development of the underlying descriptive framework was difficult. Therefore, we consider the goal of digital transformation.
- The characteristics of the experimental group lead to different challenges. Patients with stroke often have limitations related to their cognition, perception, and movement (motor function). This leads, on the one hand, to recruitment challenges owing to lower participation possibilities and lower response rate and, on the other hand, to challenges in the design, layout, and formulation of the questionnaire. Furthermore, pandemic-related inaccessibility was expected.
- Limited financial resources limit the additional number of patients with stroke that can be enrolled via a panel.
- Owing to the recruitment challenges, we decided to include a control group (the general population). Because of the scale effects, the coefficients will not be compared directly. The comparison will be made by considering the influence of the covariate general population.
- Analysis of preference differences between the experimental and control groups will be limited because financial resources do not allow adequate number of patients with stroke to be available via a panel survey.

Another limitation relates to the interpretation of the concept of “value.” The term “value” is a broad term used in different contexts. In general, “value” refers to the worth, importance, or usefulness assigned to something based on individual perceptions. However, “value” can be determined by a wide range of terms, such as personal beliefs, societal norms, economic considerations, and preferences. The economic perspective considers the exchangeable value of goods or services. In contrast, “value” can also compass moral, ethical, or philosophical principles that guide individuals’ behavior. Therefore, it is crucial to emphasize at this point that there is a potential limitation in this paper where the term “value” may be subject to misunderstanding. In this study, the concept of “value” is distinguished from “value judgments”. The term “value judgments” describes the context of ethical, moral, or philosophical aspects [71]. In the context of HPR, “value refers to the subjective assessment of the importance, desirability, or utility individuals assign to specific health-related outcomes or interventions.” It involves understanding and measuring the preferences of individuals when making decisions related to their health and health care. In contrast to judgments, preferences relate to individual choices that are based on subjective likes or dislikes. This is unclear as is. Do you mean “The guiding principle of HPR is encapsulated in the mantra “choice defines value.” This concept highlights the importance of gaining a deeper understanding of patients' preferences, enabling health care providers, regulators, and policy makers to better address the needs of patients? Please revise as applicable [72,73]. As preferences can be influenced by judgments, social determinants, environmental factors, or experiences, we included several questions in the final survey instrument that could be mapped to these influencing factors (eg, cultural background, experiences in therapy, experiences with digital health, and attitudes toward digitization). These questions can be consulted when analyzing the differences between patient groups.

### Conclusions

This HPR study provides information on preferences to analyze the criteria that impact acceptance and the value of innovative interventions using digital technologies. Developers, health care providers, and policy makers often make difficult decisions about development, reimbursement, and the choice of the benefit-maximizing intervention for each patient (group). These decisions require information on the value of clinical and nonclinical criteria derived from patient preference information. As patients are the ones who ultimately experience the positive and negative outcomes of treatment, decisions related to health care intervention options should be patient centered and reflect patient values. This is where HPR comes in, providing information about patient values relevant to decision-making [16,17]. This study will inform decision makers about the factors impacting patient and public acceptance. Understanding acceptance will improve decisions and investigations of the development, adoption, and pricing of innovative digital interventions. Furthermore, the aim of reporting the development of this DCE is to represent the standards of DCE development, provide reproducibility to verify the results, ensure transparency and ethical considerations, and improve the efficiency of the research. This protocol offers a transparent, practical, and scientific approach for eliciting patient preferences.

## Appendix

Multimedia Appendix 1 Visual representations of the levels of each attribute.

## Abbreviations

AI: artificial intelligence
DCE: discrete choice experiment
E-BRAiN: evidence-based robotic assistance in neurorehabilitation
HPR: health preference research

## Data Availability

The data sets generated and analyzed during this study are available from the corresponding author upon reasonable request.

## References

[1] Platz T, Owolabi M, Platz T (2021). Clinical pathways in stroke rehabilitation: background, scope, and methods. Clinical Pathways in Stroke Rehabilitation: Evidence-based Clinical Practice Recommendations.

[2] Vanoglio F, Bernocchi P, Mulè C, Garofali F, Mora C, Taveggia G, Scalvini S, Luisa A (2017). Feasibility and efficacy of a robotic device for hand rehabilitation in hemiplegic stroke patients: a randomized pilot controlled study. Clin Rehabil.

[3] Bertani R, Melegari C, De Cola MC, Bramanti A, Bramanti P, Calabrò RS (2017). Effects of robot-assisted upper limb rehabilitation in stroke patients: a systematic review with meta-analysis. Neurol Sci.

[4] GBD 2016 Stroke Collaborators (2019). Global, regional, and national burden of stroke, 1990-2016: a systematic analysis for the Global Burden of Disease Study 2016. Lancet Neurol.

[5] Stroke Unit Trialists' Collaboration (2013). Organised inpatient (stroke unit) care for stroke. Cochrane Database Syst Rev.

[6] Dohle CH, Quintern J, Saal S, Stephan KM, Tholen R, Wittenberg H (2015). S2e-Leitlinie »Rehabilitation der Mobilität nach Schlaganfall (ReMoS)« Kurzfassung der Konsensusversion. Neurol Rehabil.

[7] Platz T (2019). Evidence-based guidelines and clinical pathways in stroke rehabilitation-an international perspective. Front Neurol.

[8] Kolominsky-Rabas PL, Heuschmann PU, Marschall D, Emmert M, Baltzer N, Neundörfer B, Schöffski O, Krobot KJ (2006). Lifetime cost of ischemic stroke in Germany: results and national projections from a population-based stroke registry: the Erlangen Stroke Project. Stroke.

[9] Matamala-Gomez M, Bottiroli S, Realdon O, Riva G, Galvagni L, Platz T, Sandrini G, De Icco R, Tassorelli C (2021). Telemedicine and virtual reality at time of COVID-19 pandemic: an overview for future perspectives in neurorehabilitation. Front Neurol.

[10] Zsiga K, Edelmayer G, Rumeau P, Péter O, Tóth A, Fazekas G (2013). Home care robot for socially supporting the elderly: focus group studies in three European countries to screen user attitudes and requirements. Int J Rehabil Res.

[11] Kraus S, Schiavone F, Pluzhnikova A, Invernizzi AC (2021). Digital transformation in healthcare: analyzing the current state-of-research. J Bus Res.

[12] Forbrig P, Bundea A, Pedersen A, Platz T, Bernhaupt R, Ardito C, Sauer S (2020). Digitalization of training tasks and specification of the behaviour of a social humanoid robot as coach. Human-Centered Software Engineering.

[13] Platz T, Seidel J, Müller A, Goldmann C, Pedersen AL (2021). THERapy-Related InterACTion (THER-I-ACT) in rehabilitation-instrument development and inter-rater reliability. Front Neurol.

[14] Mühlbacher AC, Zweifel P, Kaczynski A, Johnson FR (2016). Experimental measurement of preferences in health care using best-worst scaling (BWS): theoretical and statistical issues. Health Econ Rev.

[15] Zweifel P (2021). Preference measurement in health using experiments. Cent Eur J Oper Res.

[16] Mühlbacher AC, Bethge S, Tockhorn A (2013). Präferenzmessung im gesundheitswesen: grundlagen von discrete-choice-experimenten. Gesundh ökon Qual manag.

[17] Mühlbacher AC, Kaczynski A, Zweifel P (2014). Experimentelle präferenzmessung im gesundheitswesen mit hilfe von best-worst scaling (BWS). PharmacoEcon Ger Res Artic.

[18] Mühlbacher A, Johnson FR (2016). Choice experiments to quantify preferences for health and healthcare: state of the practice. Appl Health Econ Health Policy.

[19] Schäfer C (2020). Grundlagen der patientencompliance und adhärenz. Patientencompliance.

[20] Zhong B, Niu W, Broadbent E, McDaid A, Lee TM, Zhang M (2019). Bringing psychological strategies to robot-assisted physiotherapy for enhanced treatment efficacy. Front Neurosci.

[21] Mühlbacher VA, Bethge S (2013). Potenziale der Präferenzmessung. Gesellschaftspolitische Kommentare.

[22] James J (2013). Patient engagement. Health Affairs Health Policy Brief.

[23] Thurstone LL (1927). A law of comparative judgment. Psychol Rev.

[24] McFadden D (1974). Conditional logit analysis of qualitative choice behavior. Frontiers in Econometrics.

[25] Uncles MD, Ben-Akiva M, Lerman SR (1987). Discrete choice analysis: theory and application to travel demand. J Oper Res Soc.

[26] (2017). Allgemeine Methoden Version 5.0.

[27] Phillips KA, Johnson FR, Maddala T (2002). Measuring what people value: a comparison of "attitude" and "preference" surveys. Health Serv Res.

[28] Ryan M, Farrar S (2000). Using conjoint analysis to elicit preferences for health care. BMJ.

[29] Louviere JJ, Hensher DA, Swait JD (2000). Stated Choice Methods Analysis and Applications.

[30] de Bekker-Grob EW, Ryan M, Gerard K (2012). Discrete choice experiments in health economics: a review of the literature. Health Econ.

[31] Clark MD, Determann D, Petrou S, Moro D, de Bekker-Grob EW (2014). Discrete choice experiments in health economics: a review of the literature. Pharmacoeconomics.

[32] Bridges JF, Hauber AB, Marshall D, Lloyd A, Prosser LA, Regier DA, Johnson FR, Mauskopf J (2011). Conjoint analysis applications in health--a checklist: a report of the ISPOR Good Research Practices for Conjoint Analysis Task Force. Value Health.

[33] Reed Johnson F, Lancsar E, Marshall D, Kilambi V, Mühlbacher A, Regier DA, Bresnahan BW, Kanninen B, Bridges JF (2013). Constructing experimental designs for discrete-choice experiments: report of the ISPOR Conjoint Analysis Experimental Design Good Research Practices Task Force. Value Health.

[34] Gun SY, Titov N, Andrews G (2011). Acceptability of internet treatment of anxiety and depression. Australas Psychiatry.

[35] Davis FD (1986). A technology acceptance model for empirically testing new end-user information systems : theory and results. Massachusetts Institute of Technology.

[36] Venkatesh V, Bala H (2008). Technology acceptance model 3 and a research agenda on interventions. Decis Sci.

[37] Venkatesh V, Davis FD (2000). A theoretical extension of the technology acceptance model: four longitudinal field studies. Manage Sci.

[38] Kim H-W, Chan HC, Gupta S (2007). Value-based adoption of mobile internet: an empirical investigation. Decis Support Syst.

[39] Szinay D, Cameron R, Naughton F, Whitty JA, Brown J, Jones A (2021). Understanding uptake of digital health products: methodology tutorial for a discrete choice experiment using the bayesian efficient design. J Med Internet Res.

[40] Leigh S, Ashall-Payne L, Andrews T (2020). Barriers and facilitators to the adoption of mobile health among health care professionals from the United Kingdom: discrete choice experiment. JMIR Mhealth Uhealth.

[41] Viberg Johansson J, Bentzen HB, Shah N, Haraldsdóttir E, Jónsdóttir GA, Kaye J, Mascalzoni D, Veldwijk J (2021). Preferences of the public for sharing health data: discrete choice experiment. JMIR Med Inform.

[42] Gc VS, Iglesias CP, Erdem S, Hassan L, Peek N, Manca A (2022). Using discrete-choice experiments to elicit preferences for digital wearable health technology for self-management of chronic kidney disease. Int J Technol Assess Health Care.

[43] Phillips EA, Himmler SF, Schreyögg J (2021). Preferences for e-mental health interventions in germany: a discrete choice experiment. Value Health.

[44] Heidel A, Hagist C, Schlereth C (2021). Pricing through health apps generated data-Digital dividend as a game changer: discrete choice experiment. PLoS One.

[45] Cheung MC, Cheng S, Arciero V (2016). Assessment of value using the American Society of Clinical Oncology (ASCO) and European Society of Medical Oncology (ESMO) frameworks for novel therapies for the hematologic malignancies. Blood.

[46] Kelly RJ, Smith TJ (2014). Delivering maximum clinical benefit at an affordable price: engaging stakeholders in cancer care. Lancet Oncol.

[47] Experts in Chronic Myeloid Leukemia (2013). The price of drugs for chronic myeloid leukemia (CML) is a reflection of the unsustainable prices of cancer drugs: from the perspective of a large group of CML experts. Blood.

[48] Dockweiler C, von Hauff M, Reller A (2020). Perspektiven der Digitalisierung für das Gesundheitswesen. forum hlz. Nachhaltige Digitalisierung. Eine noch zu bewältigende Zukunftsaufgabe.

[49] Hollin IL, Craig BM, Coast J, Beusterien K, Vass C, DiSantostefano R, Peay H (2020). Reporting formative qualitative research to support the development of quantitative preference study protocols and corresponding survey instruments: guidelines for authors and reviewers. Patient.

[50] Kallio H, Pietilä A-M, Johnson M, Kangasniemi M (2016). Systematic methodological review: developing a framework for a qualitative semi-structured interview guide. J Adv Nurs.

[51] Louviere JJ, Street D, Burgess L, Wasi N, Islam T, Marley AA (2008). Modeling the choices of individual decision-makers by combining efficient choice experiment designs with extra preference information. J Choice Model.

[52] Uldry P-F, Diener CG, Severin V (2002). Using a dual response framework in choice modelling. Proceedings of the 13th Annual Advanced Research Techniques Forum.

[53] Brazell JD, Diener CG, Karniouchina E, Moore WL, Séverin V, Uldry P-F (2006). The no-choice option and dual response choice designs. Market Lett.

[54] Diener C, Orme B, Yardley D (2006). Dual response “none” approaches: theory and practice. Proceedings of the Sawtooth Software Conference.

[55] Lancsar E, Louviere J (2008). Conducting discrete choice experiments to inform healthcare decision making: a user's guide. Pharmacoeconomics.

[56] Kessels R, Jones B, Goos P (2011). Bayesian optimal designs for discrete choice experiments with partial profiles. J Choice Model.

[57] Kohlmann T (2014). [Measuring quality of life: as simple as possible and as detailed as necessary]. Z Evid Fortbild Qual Gesundhwes.

[58] van der Vaart R, Drossaert C (2017). Development of the digital health literacy instrument: measuring a broad spectrum of health 1.0 and health 2.0 skills. J Med Internet Res.

[59] Lancsar E, Fiebig DG, Hole AR (2017). Discrete choice experiments: a guide to model specification, estimation and software. Pharmacoeconomics.

[60] Mühlbacher AC, Sadler A, Lamprecht B, Juhnke C (2020). Patient preferences in the treatment of hemophilia A: a best-worst scaling case 3 analysis. Value Health.

[61] Hess S, Train K (2017). Correlation and scale in mixed logit models. J Choice Model.

[62] Döring NB (2016). Stichprobenziehung. Forschungsmethoden und Evaluation in den Sozial- und Humanwissenschaften.

[63] Berndt AE (2020). Sampling methods. J Hum Lact.

[64] Marshall D, Bridges JF, Hauber B, Cameron R, Donnalley L, Fyie K, Johnson FR (2010). Conjoint analysis applications in health - how are studies being designed and reported?: an update on current practice in the published literature between 2005 and 2008. Patient.

[65] Yang J-C, Johnson FR, Kilambi V, Mohamed AF (2015). Sample size and utility-difference precision in discrete-choice experiments: a meta-simulation approach. J Choice Model.

[66] Rahimi B, Nadri H, Lotfnezhad Afshar H, Timpka T (2018). A systematic review of the technology acceptance model in health informatics. Appl Clin Inform.

[67] Vasanthi RK, Muniandy Y, Asoghan K (2019). Digital physiotherapy intervention in health care delivery– a narrative review. INTI J.

[68] Dai T, Tayur S (2022). Designing AI‐augmented healthcare delivery systems for physician buy‐in and patient acceptance. Prod Oper Manag.

[69] Sebastian G, George A, Jackson G (2023). Persuading patients using rhetoric to improve artificial intelligence adoption: experimental study. J Med Internet Res.

[70] Sebastian G (2022). A study on metaverse awareness, cyber risks, and steps for increased adoption. Int J Secur Priv Pervasive Comput.

[71] Harvard S, Werker GR, Silva DS (2020). Social, ethical, and other value judgments in health economics modelling. Soc Sci Med.

[72] Craig BM, Lancsar E, Mühlbacher AC, Brown DS, Ostermann J (2017). Health preference research: an overview. Patient.

[73] Jakubczyk M, Craig BM, Barra M, Groothuis-Oudshoorn CG, Hartman JD, Huynh E, Ramos-Goñi JM, Stolk EA, Rand K (2018). Choice defines value: a predictive modeling competition in health preference research. Value Health.

