# Supplementary material for: Patient and Public Acceptance of Digital Technologies in Health Care: Protocol for a Discrete Choice Experiment
Source: JMIR Res Protoc. 2023 Aug 10;12:e46056. doi: 10.2196/46056 (PMC10450540; doi:10.2196/46056)
Supplement: Multimedia Appendix 1 [file resprot_v12i1e46056_app1.docx]

Multimedia appendix 1 – Table 1: Visual representations of the levels of each attribute

| **Attributes** | **Level 1** | **Level 2** | **Level 3** | **Level 4** | **Level 5** |
| --- | --- | --- | --- | --- | --- |
| Explanation and presentation of therapy exercises | Sounds and speech  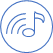 | Descriptive texts  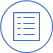 | Images  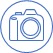 | Videos  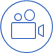 | Spatial movement  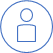 |
| Information in therapy | No information  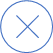 | Therapy and rehabilitation process  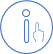 | Diagnosed disease  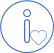 | Patient'’s current health status  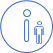 | Change in health status owing to therapy  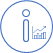 |
| Contact with health care professionals | No contact  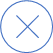 | Contact is indirect (messages)  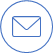 | Contact is direct (telephone or video)  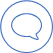 | — | — |
| Patients'’ choice in the therapy process | No influence  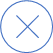 | Selection of therapy exercise with a certain degree of severity  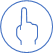 | Pace of therapy exercise  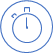 | Time of therapy (frequency, duration, and start)  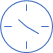 | Place of therapy (eg, home, clinic, or practice)  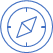 |
| Data processing | No data processing  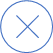 | Processing of data about the person  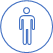 | Processing of data about the diagnosis  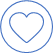 | Processing of data about the progress of the therapy  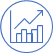 | — |
| Copayment per month | 80 € per month  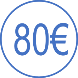 | 60 € per month  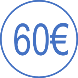 | 40 € per month  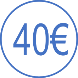 | 20 € per month  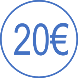 | No co-payment  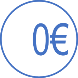 |
| Therapy success within 6 months | 60 out of 100 patients  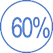 | 70 out of 100 patients  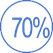 | 80 out of 100 patients  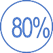 | 90 out of 100 patients  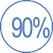 | 100 out of 100 patients  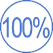 |
